# Case Report: A Fatal Case of Malignant Posterior Reversible Encephalopathy Syndrome in the Setting of Diabetic Ketoacidosis

**DOI:** 10.7759/cureus.45218

**Published:** 2023-09-14

**Authors:** Wilson Rodriguez, Shannon Tseng, Francesca Pastrana, Fajun Wang

**Affiliations:** 1 Neurology, Saint Louis University School of Medicine, Saint Louis, USA

**Keywords:** dka, pres, case report, diabetic ketoacidosis, posterior reversible encephalopathy syndrome

## Abstract

Posterior reversible encephalopathy syndrome (PRES) is a clinicoradiological syndrome that typically presents with headache, visual disturbances, seizures, and altered consciousness. Its hallmark radiological features involve subcortical white matter lesions on magnetic resonance imaging (MRI), predominantly in the parietal and occipital lobes. While generally reversible with favorable outcomes, a minority of cases may progress to malignant cerebral edema and herniation, resulting in death. We present an unusual case of a 47-year-old woman who developed malignant PRES associated with severe diabetic ketoacidosis (DKA). Despite aggressive medical and surgical treatments, the patient's condition worsened, indicating the potential for devastating outcomes in malignant PRES. This case adds to the limited body of literature that suggests the need for vigilance in monitoring patients with severe glycemic disturbances for neurological complications, such as PRES. It also highlights the importance of early recognition and aggressive management in improving neurological outcomes in malignant PRES. Further research is warranted to understand the underlying mechanisms better and identify optimal treatment strategies for this rare but potentially life-threatening condition.

## Introduction

Posterior reversible encephalopathy syndrome (PRES) is a clinicoradiological syndrome characterized by diverse clinical symptoms, including headache, visual disturbance, seizures, and impaired consciousness. The hallmark radiological findings involve subcortical white matter lesions on MRI, particularly in the parietal and occipital lobes [1]. The exact pathophysiology of PRES remains unclear, though it has been commonly associated with conditions that alter cerebral autoregulation, such as hypertension, sepsis, preeclampsia, and autoimmune disorders [2]. Despite its potential reversibility and favorable outcomes, some cases can result in devastating outcomes. This report presents an unusual case of PRES in a patient with poorly controlled type 1 diabetes mellitus (T1DM) who developed malignant cerebral edema and herniation despite maximal medical and surgical treatments.

## Case presentation

**Figure 1:**
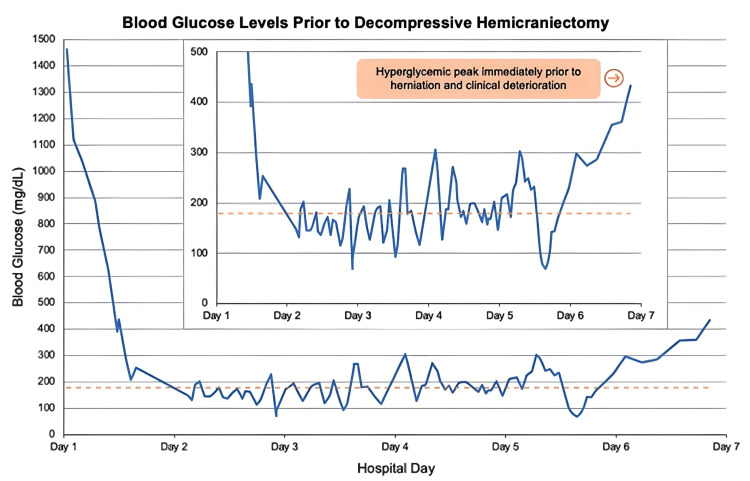
Blood glucose trend Blood glucose trend in the first seven days of hospitalization is represented by the solid line. The dotted line marks the upper (180 mg/dL) limit of the target blood glucose level.

A 47-year-old woman with a medical history of poorly controlled T1DM was brought to the emergency department (ED) after being found unresponsive at home with a blood pressure of 40/20 mmHg, heart rate of 140 beats per minute, temperature of 30.6 degrees ℃, and a "high" glucometer read (Figure 1).

The initial neurologic exam revealed lethargy and orientation to person only; no localizable deficits were identified. Laboratory studies revealed severe diabetic ketoacidosis (DKA), including a glucose level of 1,462 mg/dL (reference range 70-115 mg/dL), venous pH <6.81 (reference range 7.31-7.41), anion gap of 24mmol/L (reference range 4 to 12 mmol/L), bicarbonate <5 mEq/L (reference range 22-29 mEq/L), beta-hydroxybutyrate of 11.52 mmol/L (reference range < 0.5 mmol/L), corrected sodium level of 135 mEq/L (reference range 135-145 mEq/L), and potassium level of 7 mEq/L (reference range 3.5-4.5 mEq/L).

**Figure 2:**
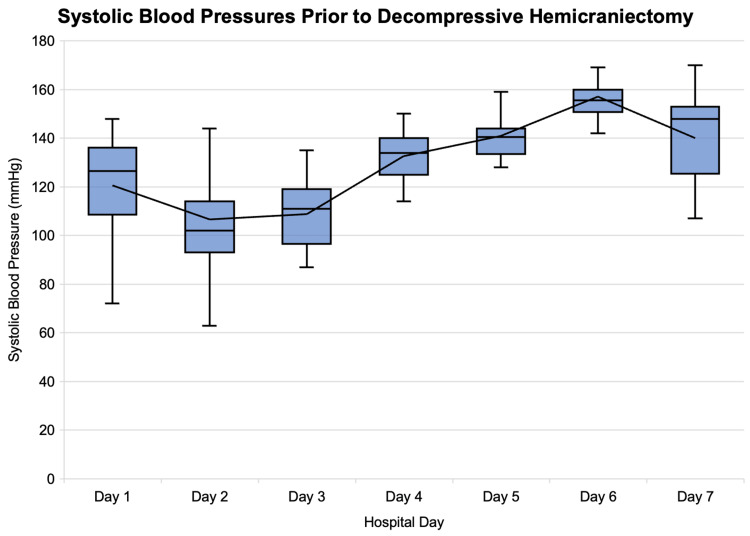
Blood pressure trend in the first seven days of hospitalization

She was immediately treated with insulin infusion and fluid resuscitation in the ED. Upon arrival to the medical intensive care unit, she was found to have agonal respirations and persistent hypoxia despite high-flow oxygen via a venturi mask. Due to this deterioration, she was emergently intubated. Subsequently, she developed refractory shock, which was attributed to a combination of hypovolemia due to severe DKA and suspected sepsis (Figure 2).

Empiric broad-spectrum antibiotics were initiated. Stress-dose steroids were also added empirically, resulting in episodes of rebound hyperglycemia. Her insulin drip was adjusted accordingly, but her blood glucose continued to fluctuate throughout the fourth day of hospitalization. Her shock rapidly resolved on the following day, and no organism was identified from urine, respiratory, and blood cultures. Despite improvement in volume status and metabolic derangements, the patient remained comatose on the exam.

**Figure 3:**
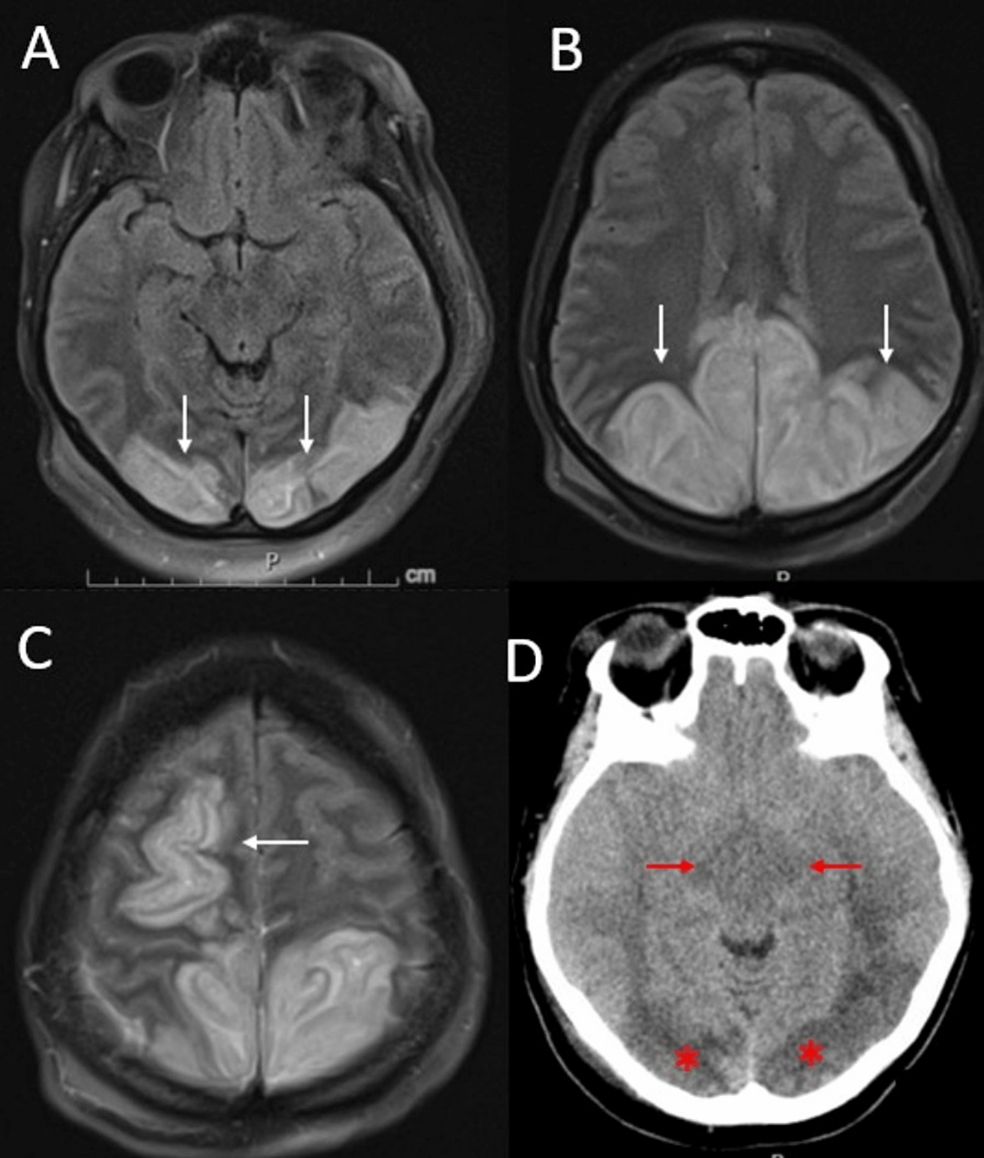
Magnetic resonance imaging and computed tomography of the brain Fluid attenuated inversion recovery sequence demonstrating hyperintensities in subcortical white matter of bilateral occipital (A, white arrows), bilateral parietal (B, white arrows), and right frontal lobes (C, white arrows). A CT on the seventh day of hospitalization demonstrates bilateral mass effect on the midbrain with effacement of ambient cisterns in axial view (D, red arrows) and persistent hypodensity following subcortical white matter of bilateral occipital lobes (D, red asterisks).

Magnetic resonance imaging (MRI) of the brain revealed edema in the bilateral parietal, occipital, and frontal lobes, as well as bilateral basal ganglia, consistent with posterior reversible encephalopathy syndrome (PRES) (Figure 3A-C).

Continuous electroencephalogram did not show any epileptiform discharges. Magnetic resonance angiography showed no signs of vascular abnormalities.

Insulin drip was weaned on the fifth day of hospitalization after the patient became hypoglycemic at 79mg/dL, and it was subsequently stopped on the sixth day of hospitalization. On the seventh day, the patient's glucose level rose to 434 mg/dl. Concurrently, she developed bradycardia and anisocoria with a dilated nonreactive right pupil. Emergent computed tomography (CT) of the head showed effacement of the bilateral ambient cisterns, indicating impending uncal herniation (Figure 3D). To address concerns of cerebral herniation, hypertonic saline was administered, leading to the resolution of anisocoria. The patient underwent emergent right decompressive hemicraniectomy (DHC) and external ventricular drain placement. Despite neurosurgical intervention and maximal medical support, she continued to have frequent intracranial pressure (ICP) crises. On the 12th day of hospitalization, a repeat CT head revealed worsening cerebral edema with herniation. After extensive family discussion, her code status was changed to comfort measures only, and she expired on the same day.

## Discussion

PRES is a clinicoradiological syndrome characterized by diverse clinical symptoms, including headache, visual disturbance, seizures, and impaired consciousness. The hallmark radiological findings involve subcortical white matter lesions on MRI, particularly in the parietal and occipital lobes [1]. While PRES is generally considered a reversible process with favorable outcomes, mortality has been reported in some cases. The term "malignant PRES" has been proposed to describe cases with permanent neurological damage or death [2-3]. Another study defined malignant PRES by following criteria: 1) a Glasgow Coma Scale (GCS) score less than 8 with "clinical decline despite standard medical management of elevated intracranial pressure" and 2) radiological evidence of edema or intracerebral hemorrhage exerting mass effect, such as effacement of basal cisterns and transtentorial, tonsillar or uncal herniation [2].

The exact pathophysiology of PRES remains unknown. In a study of 120 cases of PRES, the mean peak systolic blood pressure (SBP) was 199 mmHg, with a range of 160-268 mmHg [4]. Though not as common as hypertension, other precipitating factors that disrupt cerebral autoregulation have also been associated with PRES, such as autoimmune disease, renal failure, cytotoxic medications, sepsis, preeclampsia, and eclampsia [4-6].

Several adult cases reported previously regarding PRES attributed to severe glycemic disturbance, such as DKA and hyperosmolar hyperglycemic syndrome [7-9]. In the setting of DKA, inflammation-induced endothelial dysfunction, dysregulation of sodium-hydrogen channels, osmotic diuresis, and release of inflammatory cytokines might have contributed to the disruption of cerebral autoregulation and the development of malignant cerebral edema [10-11].

In this case, the patient presented with severe DKA with extremely high blood glucose levels and severe metabolic acidosis. Despite the initiation of appropriate treatment, including insulin drip and fluid resuscitation, her neurological status did not improve. MRI findings revealed characteristic features of PRES, with vasogenic edema in the bilateral parietal, occipital, and frontal lobes, as well as the basal ganglia. The patient experienced fluctuating blood glucose levels throughout her hospital stay and a spike in blood glucose levels preceding the development of cerebral herniation. It is plausible that DKA-impaired cerebral autoregulation, combined with wide fluctuations in blood pressure (Figure 2), contributed to the development of PRES. The fluctuation in blood glucose may have also exacerbated existing cerebral edema, leading to the progression of malignant PRES.

Early recognition and diagnosis of malignant PRES are crucial and may reduce mortality and improve neurological outcomes [1]. Treatment strategies, including aggressive neurointensive care, decompressive craniectomy, and strict intracranial pressure management, have been associated with better outcomes in malignant PRES cases [2]. This case also adds support to the existing literature on maintaining strict blood glucose control in patients with acute brain injury. Although there are no specific guidelines for glucose management in PRES, a target blood glucose range of 140 to 180 mg/dL is generally recommended for critically ill patients [12].

## Conclusions

In conclusion, this case highlights the potential for malignant PRES to occur in the setting of DKA. It underscores the importance of strict glycemic control in preventing irreversible neurological damage in patients with DKA. Early recognition and aggressive management are essential for improving neurological outcomes in patients with malignant PRES. Further research is needed to elucidate the underlying mechanisms and identify optimal treatment strategies for this rare but potentially devastating condition.
